# Characteristics of *tet*(X4)−Producing *Escherichia coli* in Chicken and Pig Farms in Hunan Province, China

**DOI:** 10.3390/antibiotics12010147

**Published:** 2023-01-11

**Authors:** Jie Yang, Gang Xiao, Ning Xiao, Zonghan Jiang, Chao Jiang, Yujuan Li, Wenxin Chen, Hongguang Lin, Zhiliang Sun, Jiyun Li

**Affiliations:** 1 College of Veterinary Medicine, Hunan Agricultural University, Changsha 410128, China; 2 Hunan Engineering Technology Research Center of Veterinary Drugs, Hunan Agricultural University, Changsha 410128, China; 3 Department of Basic Medicine, Xiangnan University, Chenzhou 423000, China

**Keywords:** multidrug resistance, plasmid, *tet*(X4), chicken, pig, *Escherichia coli*

## Abstract

Background: The plasmid−mediated tigecycline resistance gene *tet*(X4) confers a high level of resistance to tigecycline. The experiment aims to investigate the prevalence and characterization of *tet*(X4) in *Escherichia coli* isolates from chicken and pig farms in Hunan province, China. Methods: A total of six *tet*(X4) positive strains were identified in 257 *E. coli* derived from chicken samples in Xiangtan city (*n* = 2), pig samples in Xiangxiang city (*n* = 1), Chenzhou city (*n* = 2), and Zhuzhou city (*n* = 1). The presence of *tet*(X4) was directly detected by PCR assay, and then the broth dilution method determined the antimicrobial susceptibility profile of the *tet*(X4)−positive isolates. Genomic locations were identified by whole−genome sequencing (WGS) and bioinformatics. Results: Almost all *tet*(X4)−positive strains showed high resistance to multidrug, including tigecycline. Resistome analysis revealed many antibiotic resistance genes, including those with resistance to tetracyclines, β−lactams, phenicols, quinolones, lincosamides chloramphenicol, aminoglycosides and sulfamids. These *tet*(X4)−bearing strains exhibited six distract STs, such as ST10, 202, ST218, ST362, ST2077, ST7068. The plasmid replicon types carrying *tet*(X4) were the hybrid plasmid IncFIA(HI1)/IncHIA/IncHIB(R27) (5/6) and IncX1 (1/6). Conclusions: The presence of similar genetic environments in *E. coli* from different cities suggests there may be horizontal transmission pathways promoting the broad spread of drug−resistant genes in Hunan Province, putting great pressure on multidrug resistance monitoring.

## 1. Introduction

Tigecycline is regarded as a ‘last resort’ antibiotic to treat clinical infection caused by multidrug−resistant (MDR) and even extensively drug−resistant bacteria [1]. However, novel plasmid−mediated high levels of tigecycline resistance genes *tet*(X3)/*tet*(X4) were discovered in *Enterobacteriaceae* and *Acinetobacter* isolates from animals and humans in China in 2019, which has drawn worldwide attention and has posed a major threat to public health [2,3]. Since then, diverse *tet*(X) genes, ranging from *tet*(X5) [4], *tet*(X6) [5], and *tet*(X7) [6] to *tet*(X13) [7], *tet*(X14) [8], and *tet*(X15) [9], have been described. The prevalence of the *tet*(X4) gene, the most widespread *tet*(X) variant [1], has posed a great challenge to public health. Furthermore, *tet*(X4)−bearing plasmids (e.g., IncX1, IncHI2, and IncFIA) have been identified worldwide from human, poultry, food, and environmental samples [10,11,12,13,14]. Most *tet*(X4)−bearing plasmids are multidrug−resistant plasmids with various replicon types (e.g., IncX1, IncFIA, IncHIA, and IncHIB) carrying various ISs (insert sequences), especially *IS*CR2 [15]. In addition, *IS*CR2 is adjacent to *tet*(X4) in most plasmids and plays an important role in the transmission of *tet*(X4) [16]. Mobile elements with multidrug resistance genes increase the possibility of transmission in the transportation chain and further give rise to the risk of problems in public health.

Chickens and pigs are the main farmed animals in Hunan Province, with the emergence of multidrug resistance genes conferring resistance to most antibiotics in farms, bringing great pressure to daily management and limiting the use of antibiotics. In this study, we describe the characteristics and molecular epidemiology of *tet*(X4)−positive *E. coli* isolates in chicken and pig farms in Hunan Province, China, to provide experimental data and a basis for drug resistance investigation and surveillance.

## 2. Results and Discussion

### 2.1. Bacteria Isolates

A total of six isolates positive for the *tet*(X4) gene were collected from four cities located far apart, with the prevalence rate of *tet*(X4) positivity at 2.33% (6/257). These *tet*(X4)−positive strains were from chicken fecal samples from Xiangtan city (2/80) and pig fecal samples from Chenzhou city (2/75), Xiangxiang city (1/52), and Zhuzhou city (1/50), with prevalence rates of 2.5%, 2.67%, 1.93%, and 2%, respectively (Table S1). This prevalence of *tet*(X4)−positive strains are low compared to reports from chicken or pig farms in Jiangsu (18.24%) [17], Shandong (66.7%) [2], and Shanghai (12.24%) [18] in China, which may be due to the insufficient scope of the survey and the number of samples, as well as the need to remain vigilant and strengthen the surveillance in Hunan Province. According to previous reports, plasmid−mediated *tet*(X3) was detected in food samples in *Acinetobacter* from 2015–2018 in Hunan province, China. The emergence of the *tet*(X4) gene was first reported in these cities and may be attributed to daily farm management and potential transmission.

### 2.2. Antimicrobial Susceptibility Testing and Resistance Genes

**Figure 1:**
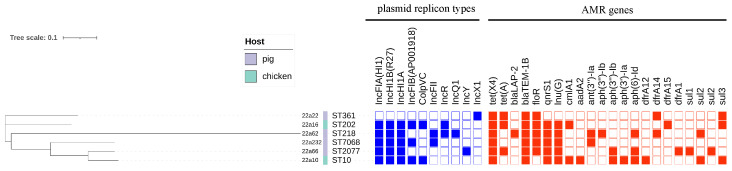
The phylogenetic tree and genomic features of six *tet*(X4)−positive *E. coli* isolates. The heatmap in different colors depicts the presence or absence of the plasmid replicon types (blue) and antimicrobial−resistance (AMR) genes (red). The whole genome sequence data of six *E. coli* strains have been submitted to NCBI under the BioProject accession number PRJNA898522.

According to MICs (minimum inhibitory concentrations) compared with the resistance point in CLSI, these *tet*(X4)−positive isolates showed resistance to multiple drugs; they were all resistant to ampicillin (≥256 mg/L), florfenicol (≥64 mg/L), gentamicin (≥64 mg/L), nalidixic (≥8 mg/L), trimethoprim–sulfamethoxazole (>16 mg/L), and tigecycline (≥4 mg/L), but susceptible to amikacin (0.5 mg/L), cefotaxime (≤0.125 mg/L), meropenem (≤0.5 mg/L), and colistin (≤0.25 mg/L) (Table 1). It is noteworthy that the MICs of these strains toward polymyxin, amikacin, and cefotaxime were not very different, but the MIC of strain 22a16 toward meropenem was four times the MIC of the other strains toward meropenem, which may indicate a trend of resistance to carbapenem antibiotics in the farm. The resistance phenotype could, in most cases, be explained by the carriage of the corresponding resistance genes. In addition, the genotype analysis of antimicrobial resistance genes (ARGs) revealed 21 ARGs for seven antimicrobial classes (beta−lactam, tetracycline, aminoglycoside, sulfonamides, phenicol, fluoroquinolone, and lincosamide) that were discovered in six strains (Figure 1). *bla*_TEM−1_ (6/6), *tet*(X4) (6/6), *floR* (5/6), *lnu*(G) (5/6), and *qnrS1* (5/6) were the most common ARGs in the six isolates. Strain 22a10, belonging to ST10, showed the most resistance genes. Interestingly, although the *bla*_TEM−1_ resistance gene existed in six strains, and the *bla*_LAP−2_ resistance gene existed in strain 22a62 in particular, these *tet*(X4)−positive strains did not show resistance to cefotaxime. Six *tet*(X4)−carrying strains exhibited high resistance to multiple classes of antibiotics, especially tigecycline, which could bring about great difficulty in clinical treatment.

### 2.3. Multilocus Sequence Typing and Plasmid Replicons

These *tet*(X4)−positive strains were sequenced through whole−genome sequencing and used for phylogenetic tree analysis and other bioinformatics analyses. The phylogenetic tree analysis revealed that six *tet*(X4)−carrying isolates had genetic diversity (Table S2) and were distributed into six distinct STs (sequence types): ST10, ST202, ST361, ST218, ST2077, and ST7068 (Figure 1). Although the isolates originated from the same locations, the ST types were all different, which could suggest that transmission of the *tet*(X4) gene was extensive in various ST types. Importantly, one *tet*(X4)−positive *E. coli*, named 22a10, belonged to ST10, which was regarded as the main ST type for transferring *tet*(X4) [1].

**Figure 2:**
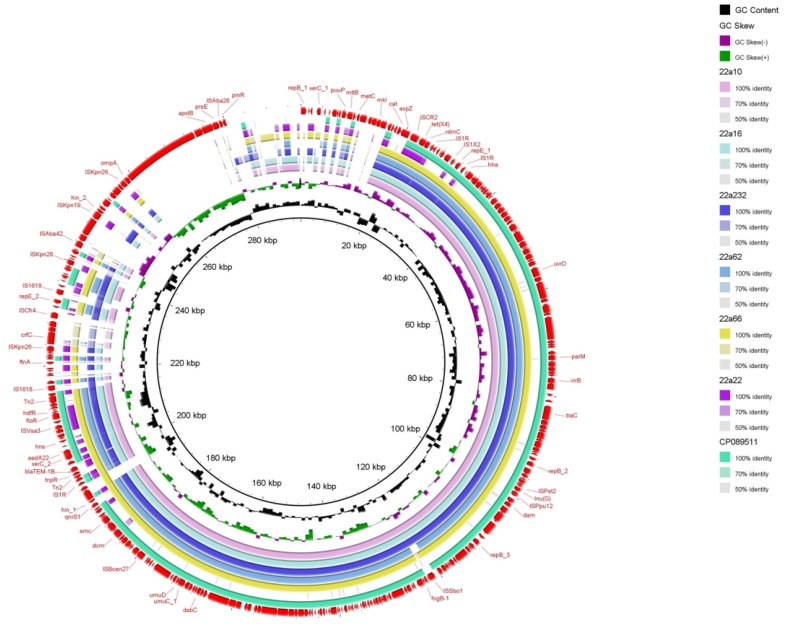
Circular comparison of the *tet*(X4)−bearing plasmids with other closely related plasmids pRT18−1 (MT219824) from the NCBI database. The outmost ring represents the reference plasmid with its gene positions.

Through bioinformatics analysis, type IV secretion systems (*vir*B) were found in almost all isolates, related to the transferability of bacteria and the contribution of *tet*(X4) to other bacteria [19] (Figure 2). The PlasmidFinder analysis of six isolates identified 10 distinct plasmid replicons: IncFIA (HI1), IncHIA, IncHIB (R27), ColpVC, IncFIB (AP001918), IncFII, IncQ1, IncR, IncY, and IncX1 (Figure 1). Interestingly, IncFIA, IncHIA, and IncHIB were identified in most of these isolates, and these plasmid replicons normally constitute a hybrid plasmid [1]. Although the whole−genome sequences of strain 22a22 containing pure IncX1 replicon alone differed significantly compared with other strains of hybrid plasmid type, they showed relatively high similarity in the upstream and downstream sequences of the *tet*(X4) and *bla*_TEM−1_ resistance genes (Figure 2). These isolates were obtained from different sources and collections while sharing the same plasmid replicon, which illustrated that the hybrid plasmid IncFIA(HI1)/IncHIA/IncHIB (R27) was the dominant *tet*(X4)−carrying plasmid replicon playing an important role in the spread of the *tet*(X4) resistance gene [15]. Importantly, the IncFIA (HI1)/IncHIA/IncHIB (R27) hybrid plasmid p22a62 shared 99% identity at 88% coverage with the plasmids pSX8G−*tet*X4 (MW940625), pSY3626 (CP059284), pT16R−1 (CP046717), and pE−T306 (CP090284) in *E. coli* SX8G, *E. coli* SY3626, *E. coli* T16R−1, and *E. coli* E−T306 derived from pork, chicken meat, pet dog, and human in China, respectively. In addition, plasmid p22a62 also showed 99% identity at 73% coverage to plasmid pHNGS471−2 (CP089511) in *Klebsiella pneumoniae* strain GD21SC417 isolated from Jiangsu Province in China (Figure 2). The high similarity of the *tet*(X4)−bearing plasmid between animals and humans indicated that the plasmid had achieved a wide distribution among different origins, and the transmission of multidrug resistance, including tigecycline resistance, required more control and monitoring to reduce the potential risk of public food health problems [20].

### 2.4. Genetic Contexts and Conjugation

**Figure 3:**
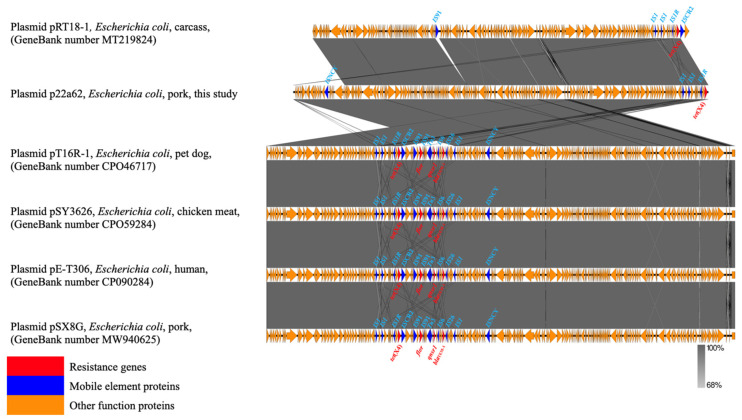
Comparison of the genetic context of *tet*(X4) with those of closely related sequences. The extent and direction of genes are shown by arrows labeled with gene names. Red arrows—resistance genes; blue arrows—mobile elements proteins; yellow arrows—other functional proteins.

Six *tet*(X4)−harboring strains showed the same genetic environment compared with the structure *IS*CR2/*tet*(X4)/*RdmC/hp*/*IS*1R of the original *tet*(X4)−carrying plasmid pRT18−1 (MT219824) (Figure 2 and Figure 3). On the one hand, *IS*CR2 was regarded as the most normal mobile element on the upstream−flanking region of *tet*(X4) [21]. On the other hand, *IS*1 mobile elements have also been reported for the transmission of *tet*(X4) [22]. The transmission of *tet*(X4) by two accustomed IS elements calls for increased monitoring, and this identical group of genetic contexts that occurred in most farms may indicate that the structure *IS*CR2−*tet*(X4)−*RdmC−hp*−*IS*1R was conducive to the spread of *tet*(X4) [22,23]. Furthermore, there were other types of mobile elements, such as *IS*Vsa3 (Figure 2), which could have mediated tet(X4) transfer in conjugation and conferred a mild fitness cost [2], and *Tn*2 transposon, which was most abundant in *bla*_TEM_−harboring strains [24].

Plasmids carrying *tet*(X4) from six strains were successfully transferred to *E. coli* C600, with transfer frequencies ranging from 10^−6^ to 10^−4^. This result indicated that the *tet*(X4) genes were located on a plasmid, and the hybrid−type replicons showed greater transfer compared with strain 22a22, which harbored IncX1−type replicons. Additionally, the horizontal dissemination of *tet*(X4) by conjugative plasmids or other mobilizable genetic elements existed in most chicken and pig farms in Hunan province, China, which may have accelerated the transmission of *tet*(X4).

In summary, this is the first time that *tet*(X4) has been detected in farms in these cities in 2021, in addition to the prevalence of *tet*(X4)−positive *E. coli* at a low percentage compared with other provinces in China. Furthermore, these *tet*(X4)−harboring strains carrying similar genetic structures with extremely similar resistance phenotypes, plasmid types, and genetic environments were seen over a wide area. In addition, these *tet*(X4)−positive *E. coli* also had very high similarity to *tet*(X4)−positive strains detected in pets, farm animals, and humans in other provinces, piqued our research interest. According to what we know, the breeders in each farm are generally fixed and have no contact with each other, and the medications used in the farms are different. We hypothesize that (1) this plasmid type and gene structure are more prevalent in and conducive to the process of drug resistance transmission, and (2) the environment acts as a major reservoir of drug resistance genes, with some of these potential transmission pathways facilitating the transmission of drug resistance genes. However, this experiment suffers from insufficient sample data and requires further investigation and research.

## 3. Materials and Methods

### 3.1. Sample Collection and tet(X) Detection

A total of 257 samples were obtained in 2021 from chicken fecal samples in Xiangtan City (*n* = 80) and pig fecal samples in Chenzhou City (*n* = 75), Xiangxiang City (*n* = 52), and Chaling City (*n* = 50). All samples were kept in an icebox and transported to the laboratory. Then, the samples were cultured on MacConkey agar (Land Bridge, Beijing, China) and incubated at 37 °C overnight. A single pink clone was randomly selected [25]. The *tet*(X4) gene was identified in all isolates by PCR assay and Sanger sequencing using the primes described previously in Table S3 [26]. Species identification was confirmed with the 16srRNA gene [27].

### 3.2. Antimicrobial Susceptibility Testing

The *tet*(X4)−positive isolates were subjected to antimicrobial susceptibility testing for 11 antimicrobial agents (tigecycline, ampicillin, amikacin, chloramphenicol, cefotaxime, nalidixic acid, florfenicol, colistin, meropenem, gentamicin, and trimethoprim–sulfamethoxazole) using the broth dilution method and interpreted according to the American Clinical and Laboratory Standards [28]. *E. coli* strain ATCC 25922 served as a quality control strain.

### 3.3. Whole−Genome Sequencing and Analysis

DNA was extracted from the *tet*(X4)−positive isolates using the TIANamp Bacteria DNA Kit DP302 (Tiangen Biotech, Beijing, China). The whole−genome sequence of strains was determined using Illumina HiSeq 2500 (Illumina, United States). The draft genome sequences of six *tet*(X4)−positive *E. coli* were assembled by Spades 3.14 [29]. The assembled genomes sequences were annotated using PATRIC3.6.9 (https://patricbrc.org/, accessed on 8 August 2022). The sequence types and plasmid replicon types were analyzed using the CGE server (https://cge.cbs.dtu.dk/services/), and the phylogenetic trees were generated using Parsnp (Harvest v1.1.2, https://github.com/marbl/parsnp) and visualized using iTOL (https://itol.embl.de). Ultimately, to visualize the comparative genetic features, Easyfig v2.2.3 was used to generate linear comparison figures (http://mjsull.github.io/Easyfig).

### 3.4. Conjugation Experiment

Transferability of the *tet*(X4) gene in the *tet*(X4)−positive strain was determined by conjugation experiment using *E. coli* C600 (streptomycin−resistant strain) as the recipient strain [25]. The donor and recipient strains were diluted to the 0.5 McFarland standard in Luria–Bertani (LB) broth; they were then mixed at a ratio of 1:3 and applied to a 0.22 μm filter, followed by coculture at 37 °C for 16 h. The transconjugants were screened on Mueller–Hinton agar plates containing 2 mg/L tigecycline and 1000 mg/L streptomycin. Subsequently, the transconjugants were confirmed by PCR with the primers in Table S3. The frequencies of conjugation transfer were calculated as a function of the number of transconjugants per recipient.

## 4. Conclusions

In conclusion, we isolated six *tet*(X4)−bearing strains from chicken and pork samples from various cities in Hunan Province, China. All *tet*(X4)−carrying strains exhibited high resistance to tigecycline and conferred resistance to multiple classes of antibiotics, which would bring about great difficulty in clinical treatment. Thus, we recommend using susceptible antibiotics to treat some bacterial infections in the investigated farms. Furthermore, the diversity of MLST types showed that *tet*(X4) genes have widespread sources in *E. coli.* Moreover, this study regarded the *IS*CR2−*tet*(X4)−*RdmC−hp*−*IS*1R structure as the dominant transmission potential pathway. Further attempts to reduce the risk of multidrug resistance transmission should focus on the mechanism mediated by mobile elements. In conclusion, the incidental transmission of multidrug resistance genes requires the rational use of antibiotics and improvements in strict daily management on the farm.

## Figures and Tables

**Table 1:** The MIC of 6 *tet*(X4)−positive *E. coli* strains.

Strain	Antimicrobial Agents (mg/L) ^a^										
	TGC	AMP	AMK	CPL	CTX	NAL	FFC	COL	MEM	GEN	STX
22a10	**4**	**>256**	0.5	**256**	0.06	**>256**	**128**	0.25	0.06	**>128**	**>16**
22a16	**8**	**>256**	0.5	**128**	0.06	**32**	**128**	0.125	0.5	**>128**	**>16**
22a22	**8**	**>256**	0.5	**256**	0.06	**>256**	**128**	0.125	0.06	**64**	**>16**
22a62	**4**	**>256**	0.5	**128**	0.125	**>256**	**64**	0.125	0.03	**>128**	**>16**
22a66	**8**	**256**	0.5	**128**	0.06	**64**	**128**	0.125	0.06	**>128**	**>16**
22a232	**4**	**>256**	0.5	**256**	0.125	**8**	**128**	0.125	0.06	**>128**	**>16**

^a^ Abbreviations and resistance breakpoints: TGC—tigecycline (R > 2 mg/L), AMP—ampicillin (R > 8 mg/L), AMK—amikacin (R > 16 mg/L), CPL—chloramphenicol (R > 8 mg/L), CTX—cefotaxime (R > 2 mg/L), NAL—nalidixic acid (R > 4 mg/L), FFC—florfenicol (R > 16 mg/L), COL—colistin (R > 2 mg/L), MEM—meropenem (R > 8 mg/L), GEN—gentamicin (R > 4 mg/L), SXT—trimethoprim−sulfamethoxazole (R > 4 mg/L). Bold formatting indicates resistance to the respective antimicrobial agents.

## Data Availability

The whole genome sequence data of six *E. coli* strains have been submitted to NCBI under the BioProject accession number PRJNA898522.

## Supplementary Materials

The following supporting information can be downloaded at: https://www.mdpi.com/article/10.3390/antibiotics12010147/s1, Table S1: The information of six *tet*(X4)-positive *E. coli* isolates; Table S2: The snps in six *tet*(X4)-positive strains.; Table S3: The prime that was used.
