# Supplementary material for: Characteristics of tet(X4)−Producing Escherichia coli in Chicken and Pig Farms in Hunan Province, China
Source: Antibiotics (Basel). 2023 Jan 11;12(1):147. doi: 10.3390/antibiotics12010147 (PMC9854778; doi:10.3390/antibiotics12010147)
Supplement: Supplementary file 1 [file antibiotics-12-00147-s001.zip › antibiotics-2093910-supplementary.pdf]

Table S1. The information of six *tet*(X4)-positive *E. coli* isolates.

Strain	Species	Source	Location	Transfer Frequency
22a10	*Escherichia coli*	chicken	Xiangtan City	10^{-4}
22a16	*Escherichia coli*	chicken	Xiangtan City	10^{-4}
22a22	*Escherichia coli*	pig	Xiangxiang City	10^{-6}
22a62	*Escherichia coli*	pig	Chenzhou City	10^{-4}
22a66	*Escherichia coli*	pig	Chenzhou City	10^{-5}
22a232	*Escherichia coli*	pig	Zhuzhou City	10^{-5}

Table S2. The snps in six *tet*(X4)-positive strains. The values in each row crossed with each column in the table represent the snps from which the two strains differ.

	22a10	22a16	22a22	22a232	22a62
22a10	0	29998	30907	24381	46156
22a16	29998	0	24653	29253	45070
22a22	30907	24653	0	29824	44728
22a232	24381	29253	29824	0	45505
22a62	46156	45070	44728	45505	0

Table S3. The prime that was used. *tet*(X)-F/*tet*(X)-R primers were used for identification of *tet*(X4) resistance gene. ERIC1 and ERIC2 primers were used for identification of transconjugant.

Primer	Sequence (5′-3′)	Size (bp)
tet(X)-F	CCGTTGGACTGACTATGGC	475
tet(X)-R	TCAACTTGCGTGTCGGTAA	
ERIC1	ATGTAAGCTCCTGGGGATTCAC	100-2000
ERIC2	AAGTAAGTGACTGGGGTGAGCG	
